# Developmental Remodeling of the Auditory Hair Cell Cuticular Plate Defines Transient and Mature Structural Domains

**DOI:** 10.3390/cells15070574

**Published:** 2026-03-24

**Authors:** Ai Liu, Shang Gao, Yilin Du, Zhilin Dou, Weiqing Liu, Sihao Xu, Wenjie Sun, Xi Li, Jiangxia Li, Qiji Liu, Yecheng Jin

**Affiliations:** 1 Key Laboratory for Experimental Teratology of the Ministry of Education and Department of Medical Genetics, School of Basic Medical Sciences, Cheeloo College of Medicine, Shandong University, Jinan 250012, China; 2 NHC Key Laboratory of Cardiopulmonary Rehabilitation and Functional Recovery and School of Health and Life Sciences, University of Health and Rehabilitation Sciences, Qingdao 266113, China

**Keywords:** cuticular plate, hair cell, development, Gαi-GPSM2, cytoskeleton

## Abstract

The cuticular plate, a dense F-actin meshwork anchoring stereocilia in auditory hair cells (HCs), undergoes dynamic remodeling during development, but its structural transitions remain poorly understood. Here, we identified two distinct structural domains associated with this maturation. First, a transient F-actin-free area emerges within the lateral periphery of the developing cuticular plate, presenting as a crescent-shaped region that disappears upon HC maturation. Second, the lateral margin of the mature cuticular plate itself remodels into a persistent step-like structure, exhibiting cell-type-specific geometries in inner versus outer HCs. The consistent coincidence between Gαi-GPSM2 complex disruption and aberrant development of both structures in mutant mice implies a role for this complex in their formation. Additionally, microtubules spatially complemented F-actin distribution, suggesting coordinated cytoskeletal regulation. These findings revealed a sophisticated developmental program for cuticular plate maturation.

## 1. Introduction

Auditory hair cells (HCs), located in the organ of Corti within the cochlea, are fundamental to auditory function. They are classified into inner hair cells (IHCs) and outer hair cells (OHCs), which play distinct yet complementary roles in sound processing. IHCs primarily mediate auditory transduction, whereas OHCs amplify and fine-tune these mechanical vibrations, enhancing hearing sensitivity and frequency discrimination [1]. HCs are characterized by two key apical structures: the cuticular plate and the stereocilia bundle, both composed of specialized, interconnected actin filament networks [2,3]. The stereocilia bundle, arranged in a staircase-like pattern on the HC apical surface, contains mechanotransduction complexes at its tips that convert mechanical stimuli into electrical signals [4,5]. The stereocilia rootlets insert into the underlying cuticular plate, a dense F-actin matrix [6,7,8].

Extensive research has elucidated the morphogenesis and function of the stereocilia bundle [4,9,10,11,12], the molecular composition and significance of the cuticular plate remain poorly understood. The cuticular plate is speculated to serve as a mechanical foundation for the HC apical surface, providing structural support and anchoring stereocilia [2,13,14,15]. Additionally, the gaps in the F-actin mesh along the peripheral edges of the cuticular plate could provide routes for vesicular transport [16]. Consequently, a growing number of studies have focused on elucidating the molecular mechanisms that regulate cuticular plate morphology, directly linking its structural integrity to hearing function [17,18,19,20,21,22].

Current understanding of cuticular plate development remains limited. Two models have been proposed to explain its early developmental process [23]. The first, based on ultrastructural studies [24,25], suggests that F-actin grows around developing stereocilia rootlets, intertwining to form the cuticular plate, which matures alongside the rootlets. The second model, proposed by Nishida et al. [26], posits that cuticular plate components initially assemble as a peripheral ring, consolidating into a central disk before expanding outward to form the mature structure by postnatal days (P) 3–5. Both models imply structural plasticity of the cuticular plate during development, allowing the stereocilia and their associated rootlets to adjust spatially until they reach their final size and position. Supporting this, the cuticular plate transitions from a loosely organized mesh to a highly condensed structure [13]. In mouse OHCs, the thickness of the cuticular plate increases by 66% between P0 and P5 [27]. Additionally, the apical circumference of OHCs transforms from a rounded hexagon to a mature non-convex triangle-like shape during the postnatal development [28].

Thus, while the cuticular plate is recognized as a dynamic and essential structure, the precise spatial and temporal events governing its maturation remain opaque. In this study, we further investigated cuticular plate morphogenesis, uncovering novel structural properties that enhance our understanding of its dynamic maturation process.

## 2. Materials and Methods

### 2.1. Mice

All animal experimental procedures were approved by the Ethics Committee of the School of Basic Medical Sciences, Shandong University (approval No. ECSBMSSDU2023-2-8; approval date: 24 February 2023). The animals were housed under standard conditions at 22 ± 1 °C with a 12-h light/dark cycle and were provided free access to food and water throughout the study. *Brg1^flox^*^/*flox*^ [29] and *Atoh1-Cre* [30] mouse lines were maintained on a mixed genetic background and genotyped as previously described. *Atoh1-Brg1^f/f^* [31] mice were generated as previously described. The spontaneous mutant mouse line was maintained on a C57BL/6J background. These mice exhibit profound hearing loss accompanied by apical HC morphological defects and a near-complete loss of the Gαi-GPSM2 complex at the HC apical surface. The causative genetic mutation is under investigation in a separate study and is not detailed here.

For timed pregnancies, the day of vaginal plug detection was regarded as Embryonic day (E) 0.5 and the day of birth as P0. For each developmental time point and experimental condition, at least three animals were analyzed. For each immunostaining experiment, at least 20 HCs from each cochlear turn (apical, middle, basal) were examined per condition. For electron microscopy, at least 6 HCs were analyzed per sample.

### 2.2. Immunofluorescence Staining

Cochleae were dissected and fixed via perilymphatic perfusion with 4% paraformaldehyde (PFA) in phosphate-buffered saline (PBS), followed by immersion in fixative at 4 °C overnight. The cochleae older than P5 were decalcified overnight in 10% EDTA at room temperature (RT). For whole-mount immunostaining, the sensory epithelium was dissected and divided into the apical, middle, and basal parts. Samples were blocked for 30 min with 10% donkey serum, followed by incubation with primary antibodies in PBS at 4 °C overnight. After three washes with PBS, samples were incubated at 37 °C for 1 h in secondary antibodies. F-actin filaments were visualized using phalloidin, and 4′,6-diamidino-2-phenylindole (DAPI) was used to stain the nuclei.

The following primary antibodies were used for immunostaining: anti-TRIOBP (rabbit, 1:400, 16124-1-AP, Proteintech, Wuhan, China), anti-βII-Spectrin (mouse, 1:400, 612562, BD, San Jose, CA, USA), anti-LMO7 (mouse, 1:200, sc-376807, Santa Cruz, Dallas, TX, USA), anti-SNX9 (rabbit, 1:400, 15721-1-AP, Proteintech, Wuhan, China).

Images were acquired using an LSM 880 laser scanning microscope (ZEISS, Oberkochen, Germany) equipped with an Airyscan. Line-scan fluorescence intensity analysis was performed using ImageJ (v.1.8.0).

### 2.3. Electron Microscopy

Cochleae were dissected and fixed via perilymphatic perfusion with 2.5% glutaraldehyde in PBS, followed by immersion in fixative at 4 °C overnight. The cochleae were then dissected to expose the organs of Corti and were post-fixed for 2 h in 1% osmium tetroxide. For SEM, cochleae were dehydrated in a series of graded ethanol washes, critical-point dried, mounted on metal stubs, and sputter-coated with gold. The samples were imaged using a QUANTA FEG 250 scanning electron microscope (FEI Company, Hillsboro, OR, USA) at 5 kV. For transmission electron microscopy, the samples were embedded in Epon 812 resin (SPI-Chem, West Chester, PA, USA) after post-fixation, cut into ultrathin sections (thickness, 70 nm) using an ultramicrotome, placed on copper grids, and examined using a JEOL-1200EX electron microscope (JEOL, Tokyo, Japan) at 80 kV.

## 3. Results

### 3.1. Initial Formation of the Cuticular Plate

**Figure 1:**
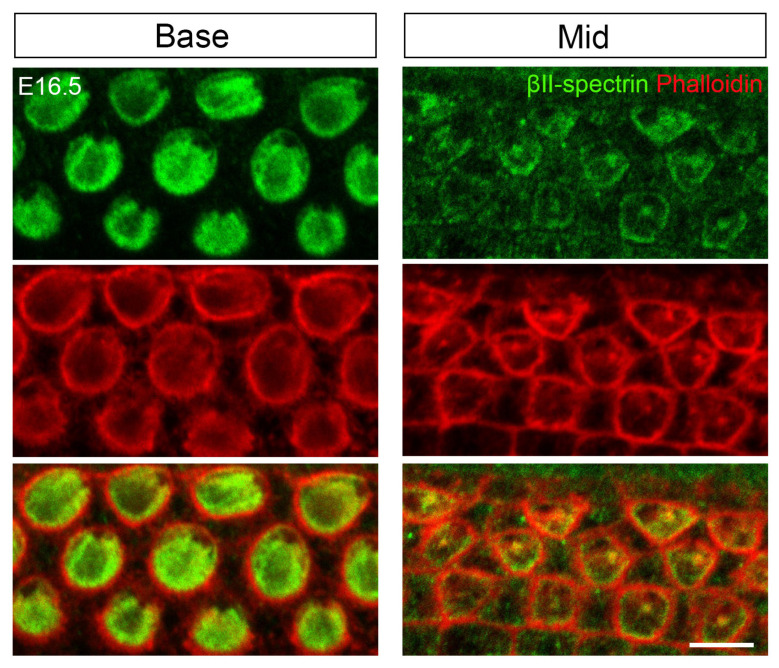
Initial morphological development of the cuticular plate. Whole-mount images of the E16.5 cochlea stained for phalloidin and βII-spectrin. Scale bar: 5 µm.

To determine which model accurately describes early cuticular plate formation, we performed immunofluorescence assays using cuticular plate-specific markers. The base-to-apex differentiation gradient along the cochlear axis allowed simultaneous observation of multiple developmental stages within individual cochleae. At E16.5, in most neonatal OHCs from the middle cochlea, βII-spectrin primarily labeled both the circumferential ring and the apical center (Figure 1). A small subset of OHCs showed labeling only in the circumferential ring (Figure 1). Phalloidin strongly stained apical junctions and the apical center but showed no significant enrichment in the circumferential ring (Figure 1). In the basal turn, cuticular plates were intensely labeled by βII-spectrin, though the original ring remained visible laterally (Figure 1). Phalloidin-labeled F-actin filled the cuticular plate, with reduced signal adjacent to the lateral ring (Figure 1). These findings demonstrate that different cuticular plate components follow distinct formation processes. F-actin organization supports the ultrastructure-based model [24,25], while βII-spectrin distribution aligns with peripheral consolidation model described by Nishida et al. [26].

### 3.2. A Transient F-Actin-Free Area Emerges Within the Lateral Periphery of the Cuticular Plate During HC Development

**Figure 2:**
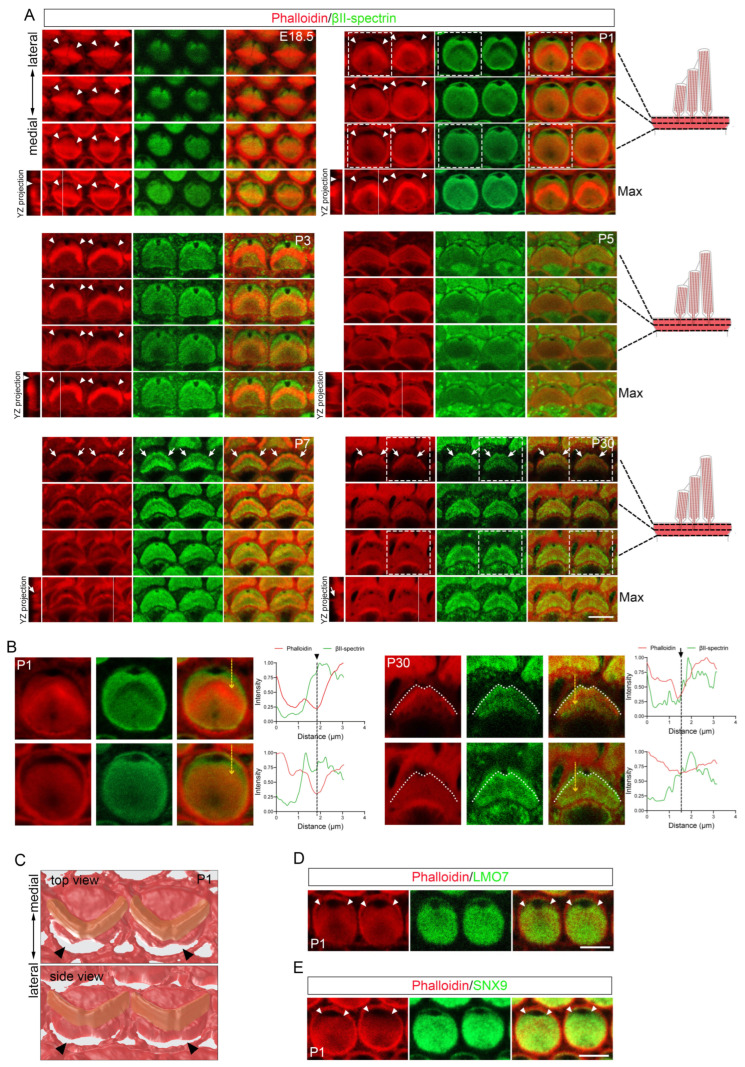
Morphological development of the OHC cuticular plate. (**A**) Whole-mount images of three depth levels and maximum intensity projections of OHC from basal cochlea at E18.5, P1, P3, P5, P7 and P30, stained for phalloidin and βII-spectrin. (**B**) Enlarged images of the dashed boxed areas in the P1 and P30 panels in (**A**). Intensity profiles from the corresponding yellow dashed arrows are shown right. White dashed lines outline the upper edge of the LSCP (the lateral margin of the cuticular plate’s apical surface). (**C**) Three-dimensional apical reconstruction of OHCs from the basal cochlea, based on phalloidin staining. (**D**) Whole-mount images of P1 OHCs from basal cochlea stained for phalloidin and LMO7. (**E**) Whole-mount images of P1 OHCs from basal cochlea stained for phalloidin and SNX9. Arrowheads indicate the TLFFA. Arrows indicate the LSCP. Scale bars: 5 µm.

Following initial formation, the cuticular plate increased in thickness [27] and showed enhanced phalloidin labeling density (Figure 2A). However, we observed a distinct lateral region where phalloidin staining not only failed to intensify but was nearly absent (Figure 2A). Whole-mount staining at E18.5 revealed a weakly phalloidin-stained, thin crescent-shaped area in the distal-abneural cortical domain of OHCs, localized to the kinocilium side in the basal cochlea (Figure 2A). By P1, this region expanded, with F-actin becoming almost undetectable (Figure 2A,B). The F-actin-free fonticulus was situated at the midpoint of the crescent and protruded medially from the crescent’s contour (Figure 2A,B). Confocal Z-stack imaging and 3D reconstruction confirmed that this F-actin-free zone spanned the entire cuticular plate along the apical-basal axis (Figure 2A,C). In the basal cochlea, the F-actin-free area diminished after P1 and disappeared entirely by P5, except for the persistent fonticulus (Figure 2A). Phalloidin staining alone cannot define the precise boundary of the cuticular plate, as F-actin is also present in the adjacent submembrane cortex. Therefore, to unambiguously determine whether this region lies within or outside the cuticular plate, we employed specific molecular markers to delineate its border. We co-stained whole-mount cochleae for phalloidin and cuticular plate markers βII-spectrin [32], SNX9 [33] and LMO7 [18]. Immunostaining of P1 OHCs from the basal cochlea confirmed that the F-actin-free area was entirely embedded within the domain defined by these markers (Figure 2A,B,D,E). This localization identifies it as a specialized, transient feature within the developing cuticular plate. Notably, within the broader F-actin-free zone, all three cuticular plate markers delineated a smaller, overlapping domain of weak or absent expression. Thus, the region deficient in cuticular plate markers constituted only a portion of the larger F-actin-free area (Figure 2A,B,D,E).

**Figure 3:**
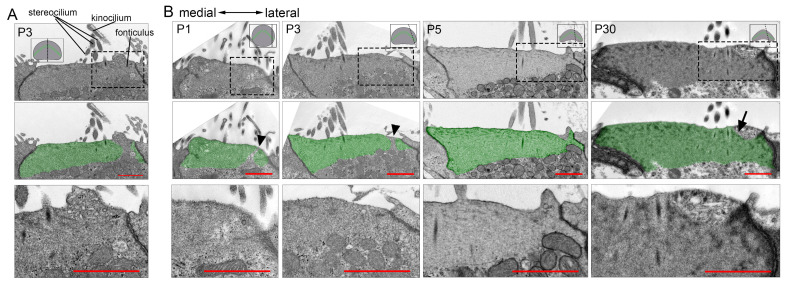
Ultrastructural analysis of OHC cuticular plate development by TEM. (**A**) TEM images of P3 OHC cuticular plate sections crossing the fonticulus from the basal cochlea. (**B**) TEM images of P1, P3, P5, and P30 OHC cuticular plate sections (non-fonticulus regions) from the basal cochlea. The electron-dense region of the cuticular plate is highlighted in green. Arrowheads indicate the TLFFA. Arrows indicate the LSCP. The bottom row of images show enlarged views of the areas within the dashed boxes in the top row. Scale bars: 1 µm.

**Figure 4:**
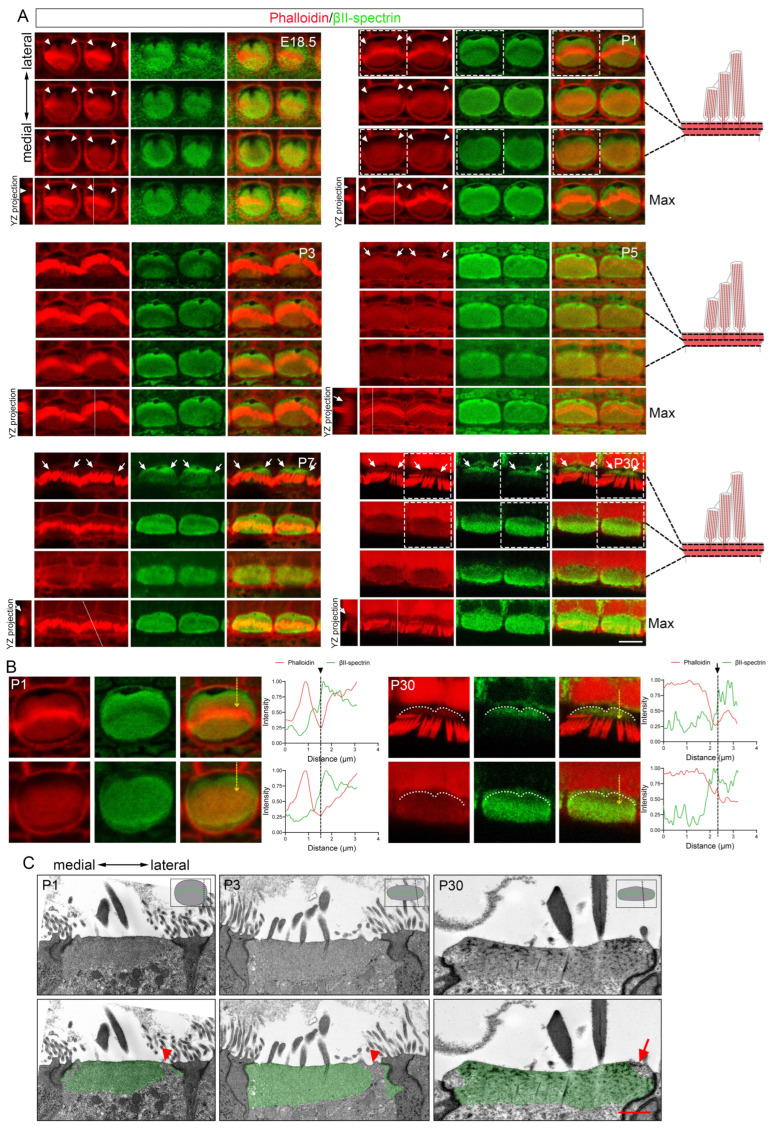
Morphological development of IHC cuticular plate. (**A**) Whole-mount images of three depth levels and maximum intensity projections of IHC from basal cochlea at E18.5, P1, P3, P5, P7, and P30, stained for phalloidin and βII-spectrin. Scale bar: 5 µm. (**B**) Enlarged images of the dashed boxed areas in the P1 and P30 panels in (**A**). Intensity profiles from the corresponding yellow dashed arrows are shown right. White dashed lines outline the upper edge of the LSCP (the lateral margin of the cuticular plate’s apical surface). (**C**) TEM images of IHC cuticular plates from basal ochlea at P1, P3, and P30. The electron-dense region of the cuticular plate is highlighted in green. Scale bar: 1 µm. Arrowheads indicate the TLFFA. Arrows indicate the LSCP.

Transmission electron microscopy (TEM) of developing OHCs revealed a low electron density zone at the fonticulus (Figure 3A), consistent with its F-actin-free nature [34]. A similar low-density region was observed near the lateral edge of the OHC apical domain, traversing the electron-dense cuticular plate in sections excluding the fonticulus at P1 and P3 (Figure 3B). In contrast to P1 and P3, a low-density region traversing the entire depth of the cuticular plate was not observed at P5 or at mature stages (Figure 3B), indicating that this particular structural arrangement ceases to exist as a traversing entity upon maturation. TEM and immunostaining also identified a comparable transient F-actin-free area in IHCs (Figure 4A–C).

**Figure 5:**
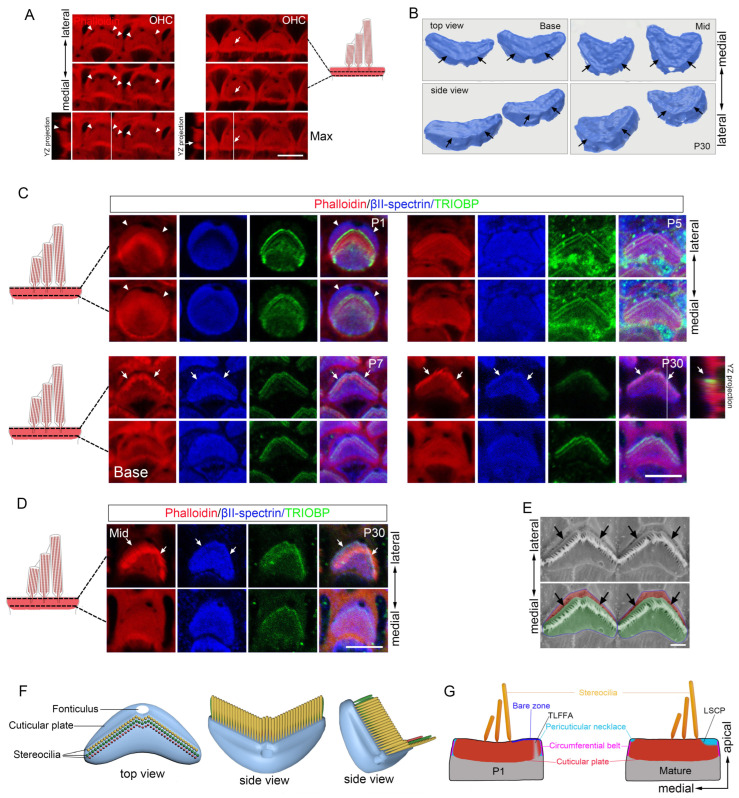
Morphological features of the LSCP in OHCs. (**A**) Whole-mount images at two depth levels and maximum intensity projections of P30 OHC from basal cochlea stained for phalloidin. Scale bars: 5 µm. (**B**) Three-dimensional reconstruction of the OHC cuticular plate from P30 basal and middle cochlea based on βII-spectrin staining. (**C**) Whole mount images of two depth levels of P1, P5, P7, and P30 OHCs from basal cochlea stained for phalloidin, βII-spectrin, and TRIOBP. Scale bars: 5 µm. (**D**) Whole-mount images of two depth levels of P30 OHC from middle cochlea stained for phalloidin, βII-spectrin, and TRIOBP. Scale bars: 5 µm. (**E**) SEM images of the mature OHC apical surfaces. The apical surface of the cuticular plate is highlighted in green, and the collapsed apical cell membrane is highlighted in red. Scale bar: 1 µm. (**F**) Three-dimensional models of the mature OHC cuticular plate from the basal cochlea. (**G**) Diagram of apical structures of the P1 and mature OHCs. Arrowheads in (**A**) indicate F-actin-free gaps at the lateral edges of HCs. Arrows in (**A**) indicate F-actin-free gaps at the medial edges of HCs. Arrowheads in (**C**) indicate TLFFA. Arrows in (**B**–**E**) indicate the LSCP. Scale bars: 5 µm. Abbreviations: TLFFA, transient lateral F-actin-free area; LSCP, lateral step of the cuticular plate.

Together, these findings demonstrate that a transient, crescent-shaped F-actin-free area (viewed from the apical surface) forms adjacent to the lateral cuticular plate edge during HC development and resolves upon maturation (schematized in Figure 5G). Although noted in prior studies [34,35], this transient lateral F-actin-free area (TLFFA) within the lateral peripheral of the cuticular plate has not been thoroughly characterized until now. The TLFFA is closely associated with the previously reported “bare zone”—a membrane-associated cortical domain characterized by polarized mInsc/GPSM2/Gαi localization and absence of microvilli/stereocilia, which spatially overlaps and aligns directly above the lateral cuticular plate [35]. Viewed apically, the TLFFA extensively overlaps with and is largely confined to a circumscribed lateral portion of this cortical domain. Critically, they reside at distinct subcellular depths: the bare zone refers to the lateral apical membrane-associated cortex, whereas the TLFFA constitutes a specialized subcompartment that projects from a specific area within this cortical domain downward into the underlying cuticular plate matrix (schematized in Figure 5G).

### 3.3. A Step-like Structure Is Formed Adjacent to the Tallest Row of Stereocilia in Mature OHC Cuticular Plates

The pericuticular necklace, an F-actin-free region between the cuticular plate and the circumferential belt [34], was confirmed to encircle the cuticular plate in mature OHCs through phalloidin and βII-spectrin co-immunostaining (Figure 2A,B). We observed that the pericuticular necklace only encircled the upper portion of the cuticular plate, while the lower region of the cuticular plate extended outward to directly interface with the circumferential belt (Figure 2A,B). In contrast to the pericuticular necklace that encircles only the upper portion of the cuticular plate, we observed F-actin-free gaps traversing the plate along its basal-apical axis at certain circumferential locations (Figure 5A), consistent with prior reports [34,36,37]. Intriguingly, the pericuticular necklace exhibited greater prominence on the lateral side compared to the medial side in mature OHCs (Figure 2A,B and Figure 5G). TEM combined with βII-spectrin immunostaining revealed that the lateral margin of the mature OHC cuticular plate forms a distinct step-like structure (Figure 2A,B, Figure 3B and Figure 5B). This composite step-like structure consists of three contiguous parts of the cuticular plate itself: (i) its horizontal apical surface, which serves as the upper platform; (ii) the lateral face of its upper portion, which descends from the platform edge; and (iii) a basal protrusion formed by its lower portion, which extends outward to directly interface with the circumferential belt (schematized in Figure 5F,G). No comparable structure was observed on the medial side. We designate this prominent lateral feature the lateral step of the cuticular plate (LSCP). Further co-immunostaining with phalloidin, βII-spectrin, and the stereocilia rootlet marker TRIOBP demonstrated that the upper edge of the LSCP, which forms the lateral margin of the cuticular plate’s apical surface, was closely opposed to the hair bundle, with its contour closely aligning with the bundle’s characteristic “V” shape (Figure 5C). The presence of the LSCP was also confirmed in mature OHCs from the middle cochlea (Figure 5D), indicating that this feature is not exclusive to the basal cochlea. Supporting these findings, scanning electron microscopy (SEM) images occasionally revealed collapsed lateral cell membranes that inadvertently highlighted the LSCP’s morphology (Figure 5E), further corroborating its existence and close association with the hair bundle.

To elucidate the developmental trajectory of the LSCP, we analyzed its formation during cuticular plate remodeling. Co-immunostaining experiments showed that the LSCP emerges between postnatal P5 and P7 in the basal cochlea (Figure 5C). TEM imaging revealed that the apical surface of the OHC cuticular plate on the lateral side of the hair bundle begins to depress or invaginate at P5 (Figure 3B). This temporal and spatial correlation suggests that the LSCP forms through a localized invagination of the apical cuticular plate surface, rather than deriving directly from the earlier TLFFA.

In summary, our study refines the architecture of the mature OHC apical domain: the pericuticular necklace encircles only its upper portion, while the lateral margin of the cuticular plate itself forms a persistent step-like structure, the LSCP, whose upper edge aligns precisely with the “V”-shaped hair bundle (schematized in Figure 5F,G). This defines a previously unrecognized structural asymmetry and specialization in cuticular plate organization.

### 3.4. The LSCP Forms Independently of the Tallest Stereocilia Row in Mature IHC Cuticular Plates

**Figure 6:**
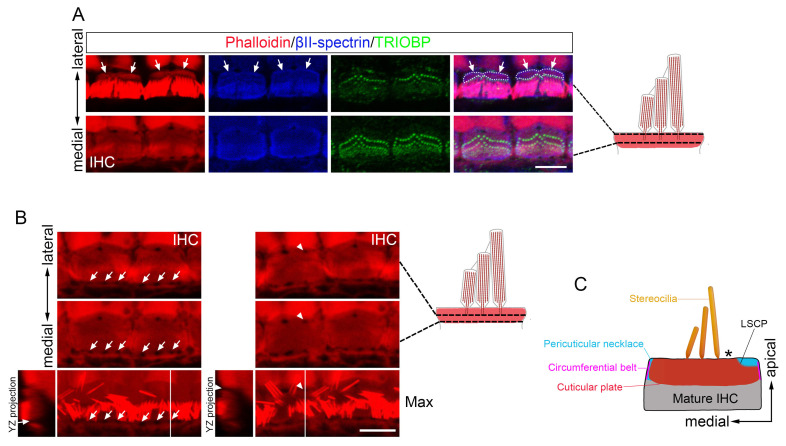
Morphological features of the LSCP in IHCs. (**A**) Whole-mount images of two depth levels of P30 IHC stained for phalloidin, βII-spectrin, and TRIOBP. Arrows indicate the LSCP. Dashed lines outline the region between the stereocilia bundle and the upper edge of the LSCP. (**B**) Whole-mount images at two depth levels and maximum intensity projections of P30 IHC stained for phalloidin. Arrowheads indicate F-actin-free gaps at the lateral edges of IHCs. Arrows indicate F-actin-free gaps at the medial edges of IHCs. (**C**) Diagram of apical structures of the mature IHCs. Asterisk indicates the spatial separation between the LSCP and the stereocilia, highlighting the primary structural difference between IHCs and OHCs. Scale bars: 5 µm. Abbreviations: LSCP, lateral step of the cuticular plate.

The LSCP was also observed in mature IHCs, though its spatial relationship to stereocilia differed significantly from that in OHCs (Figure 4A–C and Figure 6A). In OHCs, the stereocilia rootlets are positioned immediately adjacent to the upper edge of the LSCP. In contrast, the IHC LSCP maintained a distinct lateral separation from the hair bundle, with its upper edge positioned at a measurable distance from the stereocilia rootlets of the tallest stereocilia row (Figure 6A, summarized in Figure 6C). Furthermore, the LSCP in IHCs showed less precise alignment with the hair bundle compared to the tight correspondence seen in OHCs (Figure 6A). Notably, the pericuticular necklace exhibited greater prominence on the medial side of IHCs relative to OHCs (Figure 2A,B, Figure 4A and Figure 6A). However, this medial region did not form a step-like structure, as the cuticular plate extended to the cell membrane only in limited areas of the lower portion, leaving numerous intervening gaps (Figure 6B). These findings reveal fundamental differences in LSCP organization between IHCs and OHCs.

### 3.5. A Highly Specialized Complementary Distribution of Microtubules and F-Actin During Cuticular Plate Maturation

**Figure 7:**
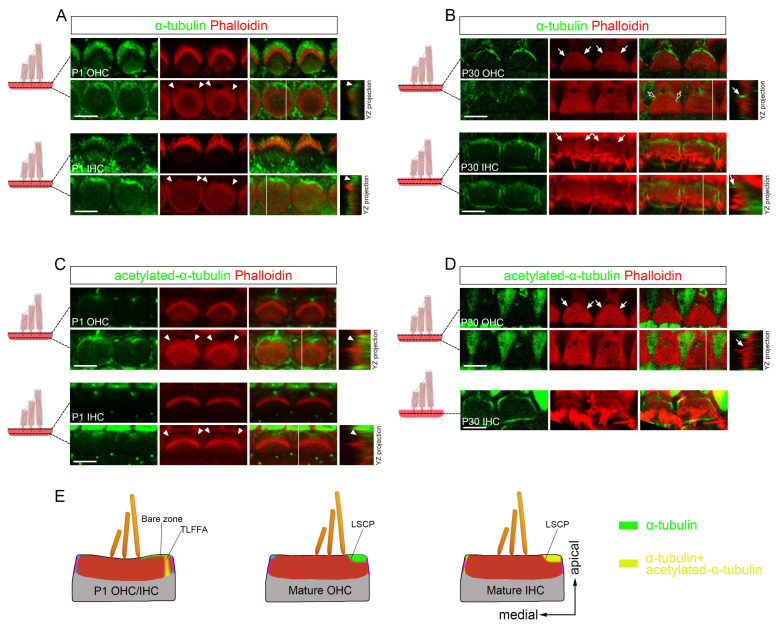
Microtubule organization at the cuticular plate level of HCs. (**A**) Whole-mount images of two depth levels of P1 OHC and IHC from basal cochlea stained for phalloidin and α-tubulin. (**B**) Whole-mount images of two depth levels of P30 OHC and IHC from basal cochlea stained for phalloidin and α-tubulin. (**C**) Whole-mount images of two depth levels of P1 OHC and IHC from basal cochlea stained for phalloidin and acetylated-α-tubulin. (**D**) Whole-mount images of two depth levels of P30 OHC and IHC from basal cochlea stained for phalloidin and acetylated-α-tubulin. (**E**) Diagram of α-tubulin and acetylated-α-tubulin localization in HCs. Arrowheads indicate the TLFFA. Arrows indicate the LSCP. Hollow arrows indicate F-actin-free gaps of the cuticular plate. Scale bar: 5 µm. Abbreviations: TLFFA, transient lateral F-actin-free area; LSCP, lateral step of the cuticular plate.

Given the central role of microtubules in cytoskeletal organization, we next examined their spatial arrangement within the HC apical cortex during cuticular plate remodeling. In developing IHCs and OHCs, microtubules exhibit a continuous distribution that extends from the apical surface into the cuticular plate. At the apical surface level, they are distributed across the entire lateral region adjacent to the stereocilia bundle—corresponding to the “bare zone” [35]. At the cuticular plate level, this microtubule array projects downward into the TLFFA. Thus, the TLFFA represents a focal downward extension of the overlying apical microtubule network (Figure 7A). In mature OHC, microtubules showed dense accumulation in the lateral region immediately adjacent to the LSCP at the upper level of the cuticular plate, an area that overlaps with the circumferential belt, while being conspicuously absent from lower portion of the cuticular plate beneath the LSCP (Figure 7B). Notably, microtubules were specifically localized within the F-actin-free gaps of the cuticular plate (Figure 7B). In mature IHCs, microtubule organization exhibited a distinct pattern with notable regional specialization. α-tubulin was primarily concentrated in the pericuticular necklace surrounding the cuticular plate periphery (Figure 7B). Laterally, microtubules concentrated adjacent to the LSCP, mirroring the OHC pattern, and extended with diminished density into the lower lateral cuticular plate (Figure 7B). Medially, in stark contrast, α-tubulin exhibited a fundamentally different organization, extending uniformly throughout the entire depth of the cuticular plate along the apico-basal axis (Figure 7B). These observations collectively demonstrate a complementary spatial relationship between microtubule and F-actin distribution in the HC apical cortex (summarized in Figure 7E).

We further characterized microtubule stability patterns by examining α-tubulin acetylation, a well-established marker of stable microtubule populations [38]. In developing HCs, acetylated-α-tubulin was detected exclusively within the TLFFA at the cuticular plate level but was absent from the overlying lateral apical cortex (the “bare zone”) where total α-tubulin is present (Figure 7C). This indicates that only the microtubules projecting into the TLFFA are stabilized during development. This principle of domain-restricted stabilization persisted in mature cells but with distinct patterns. In mature OHCs, acetylated α-tubulin showed a highly restricted localization: signal was confined to the fonticulus and was even completely absent from the lateral region immediately adjacent to the LSCP at the upper level of the cuticular plate—regions that are densely populated with total microtubules (Figure 7D). In contrast, mature IHCs exhibited acetylated α-tubulin in the pericuticular necklace, mirroring the general α-tubulin distribution pattern (Figure 7D). Collectively, these differential acetylation patterns demonstrate that microtubule stability is regulated in a precise, domain-specific manner (summarized in Figure 7E). The data further suggest that apical microtubules in mature IHCs are more stable than their OHC counterparts, which may reflect their distinct cytoskeletal demands.

### 3.6. Gαi-GPSM2 Complex Is Associated with TLFFA and LSCP Formation

Previous studies have established that the Gαi-GPSM2 complex forms a distinct thick crescent pattern at the apical cortex (the “bare zone”) lateral to the stereocilia bundle and is crucial for HC apical morphogenesis and hearing function [35,39,40,41,42]. Given this established role in a region integral to our newly identified structures, we investigated its potential involvement in the formation of the TLFFA and LSCP.

**Figure 8:**
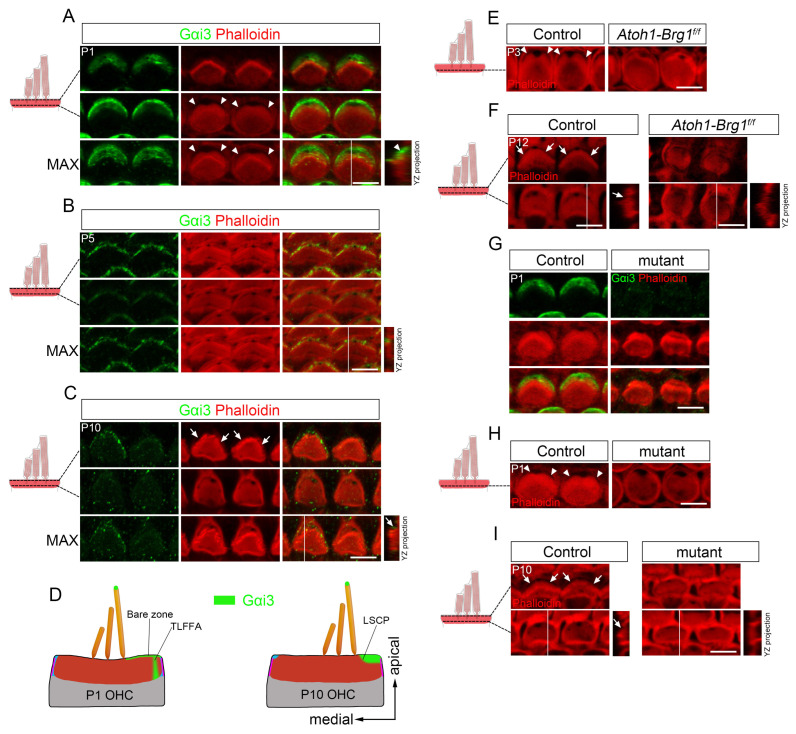
Gαi3 distribution in HCs and cuticular plate defects associated with Gαi-GPSM2 complex disruption. (**A**–**C**) Whole-mount images of two depth levels and maximum intensity projections of P1 (**A**), P5 (**B**), and P10 (**C**) OHC from basal cochlea stained for phalloidin and Gαi3. (**D**) Diagram of Gαi3 localization in OHCs. (**E**) Whole-mount images of P3 control and *Atoh1-Brg1^f/f^* OHC from middle cochlea stained for phalloidin. (**F**) Whole-mount images of two depth levels of P12 control and *Atoh1-Brg1^f/f^* OHC stained for phalloidin. (**G**) Whole-mount images of P1 control and mutant OHC stained for phalloidin and Gαi3. (**H**) Whole-mount images of P1 control and mutant OHC stained for phalloidin. (**I**) Whole-mount images of two depth levels of P10 control and mutant OHC stained for phalloidin. Arrowheads indicate the TLFFA. Arrows indicate the LSCP. Scale bar: 5 µm. Abbreviations: TLFFA, transient lateral F-actin-free area; LSCP, lateral step of the cuticular plate.

Notably, immunostaining analyses have previously revealed that the Gαi-GPSM2 complex exhibits a characteristic asymmetric distribution: its signal peaks in a focused zone at the lateral cortical domain on the kinocilium side, situated beneath a broader, weaker crescent of staining that covers the apical membrane [39]. The location and prominence of this focused zone led us to ask whether it corresponds spatially to the TLFFA identified in our study. Confocal imaging revealed a striking colocalization, with the highest intensity Gαi3 immunolabeling precisely overlapping with TLFFA in developing OHCs (Figure 8A). Furthermore, both TLFFA and the associated Gαi3 signal showed parallel developmental trajectories, with significant reductions observed by P5 (Figure 8B). In mature HCs, residual Gαi3 staining was exclusively detected in lateral region adjacent to the LSCP, mirroring the distribution of α-tubulin (Figure 8C). These findings establish a spatiotemporal correlation between Gαi3 localization and the formation of TLFFA/LSCP (summarized in Figure 8D), suggesting a potential functional role for the Gαi-GPSM2 complex in these structural specializations.

To test this hypothesis, we examined two mouse models with impaired Gαi-GPSM2 complex localization. In *Atoh1-Brg1^f/f^* mutants with aberrant Gαi-GPSM2 distribution [31], TLFFA was nearly absent in a subset of developing OHCs (Figure 8E), while LSCP formation was significantly impaired in a majority of mature HCs (Figure 8F). Complementary evidence came from a spontaneous mutant mouse line (maintained in our lab) that phenocopies Gαi-GPSM2 deficiency. This line displays a near-complete absence of the Gαi-GPSM2 complex at the HC apical surface (Figure 8G), leading to profound hearing loss and disrupted HC morphology. While the causative gene mutation is currently unpublished, the model provides an independent validation of the complex’s role. In these mutants, both TLFFA and LSCP were severely diminished in developing and mature HCs, respectively (Figure 8H,I). Although the precise mechanisms underlying Gαi-GPSM2 dysregulation in these two models remain distinct and not fully elucidated, the consistent phenotypic outcomes strongly support a close and essential link between proper Gαi-GPSM2 complex localization and the formation of both TLFFA and LSCP.

Collectively, our findings support a potential role for Gαi-GPSM2 in regulating TLFFA/LSCP formation, as evidenced by their co-localization and concurrent defects in mutant HCs. However, further studies are needed to establish a direct mechanistic link between the complex and these apical specializations.

## 4. Discussion

The cuticular plate represents a critical yet understudied structure in auditory HCs, serving dual roles as both a mechanical anchor for stereocilia bundles and a specialized compartment for mechanotransduction. Our study provides comprehensive insights into the dynamic morphological changes and molecular mechanisms underlying cuticular plate development, revealing a sophisticated, multi-stage remodeling process essential for proper HC morphology. Through detailed immunofluorescence, electron microscopy, and mutant analyses, we have characterized two previously overlooked structural features associated with cuticular plate—the TLFFA and LSCP. Although the TLFFA lacks canonical F-actin—diverging from the classical view of the cuticular plate—several lines of evidence establish it as a specialized, transient compartment within the developing cuticular plate. First, as demonstrated by co-staining with cuticular plate markers, it is strictly confined within the lateral boundary of the cuticular plate. Second, it acts as a developmental precursor, appearing before mature structures form. Third, it exhibits a distinct molecular topology: core cuticular plate markers (βII-spectrin, SNX9, and LMO7) show attenuated signals that are spatially nested within the larger F-actin-free zone, revealing an intricate sub-compartmentalization. Collectively, our work demonstrates that the cuticular plate matures through a structured sequence of domain specification, wherein transient compartments like the TLFFA guide the formation of permanent specializations like the LSCP. This paradigm fundamentally advances our understanding of how the cuticular plate transforms from a plastic primordium into the stable mechanical foundation essential for hearing.

Our immunofluorescence analysis reveals distinct assembly mechanisms between F-actin and other structural components during early cuticular plate formation. These differential developmental patterns persist in later stages, as evidenced by the TLFFA forming an expansive F-actin-free domain that consistently exceeds the boundaries of weakly labeled regions for other cuticular plate markers (βII-spectrin, SNX9 and LMO7). Intriguingly, microtubules exhibit a unique localization pattern that shows spatial complementarity with F-actin distribution across all developmental stages of the cuticular plate. Together, these findings suggest that cuticular plate development and maturation constitute a highly orchestrated process involving precise spatiotemporal coordination between F-actin, microtubules, and other associated components to mediate dynamic structural remodeling.

The observed morphological alterations in the apical circumference of HCs, particularly OHCs, cannot be explained by passive mechanisms governed by surface energy minimization, as such processes would typically yield convex (often hexagonal) boundaries. Instead, these shape changes likely involve active cellular mechanisms. Although the exact mechanisms remain to be fully elucidated, the redistribution of cortical cytoskeletal proteins—including Myosin IIA, Myosin VIIa, and Shroom2—appears to orchestrate this apical reshaping [28]. Our study demonstrates that the Gαi-GPSM2 complex localizes to the TLFFA subdomain of the developing cuticular plate and is likely critical for its formation. The diminished or aberrant localization of this complex is strongly correlated with remodeling defects in mutant models, providing compelling evidence for its role in regulating cytoskeletal dynamics during apical remodeling. This conclusion is further supported by the observation that Gαi-GPSM2 deficiency results in reduced HC apical surface area [35]. The temporal association between TLFFA formation and the remodeling process suggests that this structure may act as a developmental “adjustment zone,” enhancing cuticular plate plasticity to modulate mechanical tension and facilitate morphological transformation.

The Gαi-GPSM2 complex is known to participate in assembling effector proteins that generate mechanical forces on astral microtubules during cell division, ensuring proper mitotic spindle alignment—a mechanism evolutionarily conserved in tissues with polarized cell division [43]. In HCs, this complex is essential for apical morphogenesis and influences the organization of surface microtubules [35,39,42,44,45]. Our findings extend this understanding by revealing its persistent localization within a series of developmentally linked yet structurally distinct apical compartments. These compartments include: the “bare zone,” a broad cortical domain lateral to the kinocilium and stereocilia bundle [35]; the TLFFA, a transient, F-actin-free cytoskeletal compartment that projects downward from the “bare zone” into the developing cuticular plate, forming a specialized subdomain at the cuticular plate level; and the LSCP, the mature, step-like lateral edge of the cuticular plate. The consistent association of Gαi-GPSM2 with all three structures suggests that it functions as a continuous organizer within this lateral apical territory, coordinating the concurrent specification of the membrane-associated “bare zone” and the embedded cytoskeletal TLFFA, and ultimately the maturation of the permanent LSCP.

The close association of the Gαi-GPSM2 complex with both the TLFFA and LSCP suggests that this complex may also play a role in regulating F-actin organization within the cuticular plate. Indeed, the Gαi-GPSM2 complex has been shown to regulate F-actin dynamics in other cellular contexts. In stereocilia, it promotes F-actin bundling and is essential for establishing the characteristic staircase architecture of the hair bundle [46,47]. Similarly, in neuronal growth cones, Gαi-GPSM2 signaling modulates actin dynamics during axon guidance [47]. Interestingly, while Gαi-GPSM2 promotes F-actin polymerization in these contexts, its association with the TLFFA and LSCP—regions characterized by reduced or excluded F-actin—suggests a context-dependent functional switch. Within the developing cuticular plate, the complex may instead restrict or locally inhibit F-actin assembly, contributing to the formation of F-actin-free compartments. This apparent functional duality highlights the cell type and subcellular context specificity of Gαi-GPSM2 signaling. Whether and how the Gαi-GPSM2 complex directly regulates F-actin dynamics in the context of cuticular plate development remains to be determined in future studies.

We observed a high density of microtubules at the TLFFA, consistent with the complex’s microtubule-related functions. In mature HCs, Gαi and microtubules are also enriched in the lateral cortical region immediately adjacent to the LSCP. Given that microtubules provide structural support and compressive resistance while F-actin filaments bear tension to drive cell shape changes [48,49], the microtubules recruited by Gαi-GPSM2 may provide essential mechanical support for maintaining the transient TLFFA and later stabilizing the LSCP. Thus, the known morphogenetic functions of this complex appear to include the spatial patterning of both the transient TLFFA and the mature LSCP, translating an early developmental cue into the intricate structural asymmetry of the mature cuticular plate.

Notably, we identified striking differences in LSCP organization between IHCs and OHCs. In OHCs, the tallest rows of stereocilia are positioned in close proximity to the upper edge of the LSCP, which itself is shaped to follow the bundle’s “V” contour. In contrast, in IHCs, a consistent lateral gap separates the stereocilia insertion site from the upper edge of the LSCP, and the contour alignment is less precise, indicating a detached structural relationship. Furthermore, LSCP-associated microtubules in IHCs showed significantly higher acetylation levels compared to OHCs, suggesting greater microtubule stability in IHCs. These structural specializations likely reflect the divergent functional roles of IHCs and OHCs in auditory processing. As mechanical amplifiers, OHCs enhance sound sensitivity and frequency selectivity, while IHCs serve as primary sensory transducers, converting mechanical vibrations into neural signals [50,51,52,53]. The OHC stereocilia are mechanically coupled to the tectorial membrane, experiencing direct displacement during sound stimulation. This requires substantial structural flexibility to accommodate mechanical deformation during cochlear amplification. Conversely, IHC stereocilia lack firm tectorial membrane attachments and are presumably stimulated by viscous drag forces from endolymph movement in the subtectorial space [54,55]. The close apposition of the tallest stereocilia row to the LSCP in OHCs, along with their precise spatial coordination, suggests that the LSCP may optimize mechanical coupling and modulate stereociliary elasticity, thereby protecting against excessive deformation damage.

These findings carry significant implications for understanding auditory physiology and the mechanisms underlying hearing disorders. The molecular components and structural transitions we identified offer promising therapeutic targets for both genetic and acquired forms of hearing loss. By delineating the dynamic development of the cuticular plate, our study establishes a critical framework for future research in hair cell biology and auditory pathology.

## 5. Conclusions

This study establishes a refined developmental framework for auditory HC apical morphogenesis by identifying and characterizing two novel structural domains associated with the cuticular plate: the transient lateral F-actin-free area (TLFFA) and the lateral step of the cuticular plate (LSCP). We demonstrate that the cuticular plate matures through a precisely orchestrated sequence involving spatially complementary remodeling of F-actin and microtubule networks. The persistent association of the Gαi-GPSM2 complex with the “bare zone,” TLFFA, and LSCP positions it as a central coordinator translating early cortical patterning into stable cytoskeletal asymmetry.

## Data Availability

The data that support the findings of this study are available within the article. Any additional data required are available from the corresponding author upon reasonable request.

## References

[1] Ashmore J. (2008). Cochlear outer hair cell motility. Physiol. Rev..

[2] Drenckhahn D., Engel K., Hofer D., Merte C., Tilney L., Tilney M. (1991). Three different actin filament assemblies occur in every hair cell: Each contains a specific actin crosslinking protein. J. Cell Biol..

[3] DeRosier D.J., Tilney L.G., Egelman E. (1980). Actin in the inner ear: The remarkable structure of the stereocilium. Nature.

[4] Petit C., Richardson G.P. (2009). Linking genes underlying deafness to hair-bundle development and function. Nat. Neurosci..

[5] Schwander M., Kachar B., Muller U. (2010). Review series: The cell biology of hearing. J. Cell Biol..

[6] Itoh M. (1982). Preservation and visualization of actin-containing filaments in the apical zone of cochlear sensory cells. Hear. Res..

[7] DeRosier D.J., Tilney L.G. (1989). The structure of the cuticular plate, an in vivo actin gel. J. Cell Biol..

[8] Kitajiri S., Sakamoto T., Belyantseva I.A., Goodyear R.J., Stepanyan R., Fujiwara I., Bird J.E., Riazuddin S., Riazuddin S., Ahmed Z.M. (2010). Actin-bundling protein TRIOBP forms resilient rootlets of hair cell stereocilia essential for hearing. Cell.

[9] Goodyear R.J., Marcotti W., Kros C.J., Richardson G.P. (2005). Development and properties of stereociliary link types in hair cells of the mouse cochlea. J. Comp. Neurol..

[10] Nayak G.D., Ratnayaka H.S., Goodyear R.J., Richardson G.P. (2007). Development of the hair bundle and mechanotransduction. Int. J. Dev. Biol..

[11] Shin J.B., Krey J.F., Hassan A., Metlagel Z., Tauscher A.N., Pagana J.M., Sherman N.E., Jeffery E.D., Spinelli K.J., Zhao H. (2013). Molecular architecture of the chick vestibular hair bundle. Nat. Neurosci..

[12] Park J., Bird J.E. (2023). The actin cytoskeleton in hair bundle development and hearing loss. Hear. Res..

[13] Self T., Mahony M., Fleming J., Walsh J., Brown S.D., Steel K.P. (1998). Shaker-1 mutations reveal roles for myosin VIIA in both development and function of cochlear hair cells. Development.

[14] Tilney L.G., Cotanche D.A., Tilney M.S. (1992). Actin filaments, stereocilia and hair cells of the bird cochlea. VI. How the number and arrangement of stereocilia are determined. Development.

[15] Furness D.N., Mahendrasingam S., Ohashi M., Fettiplace R., Hackney C.M. (2008). The dimensions and composition of stereociliary rootlets in mammalian cochlear hair cells: Comparison between high- and low-frequency cells and evidence for a connection to the lateral membrane. J. Neurosci..

[16] Kachar B., Battaglia A., Fex J. (1997). Compartmentalized vesicular traffic around the hair cell cuticular plate. Hear. Res..

[17] Liu Y., Qi J., Chen X., Tang M., Chu C., Zhu W., Li H., Tian C., Yang G., Zhong C. (2019). Critical role of spectrin in hearing development and deafness. Sci. Adv..

[18] Du T.T., Dewey J.B., Wagner E.L., Cui R., Heo J., Park J.J., Francis S.P., Perez-Reyes E., Guillot S.J., Sherman N.E. (2019). LMO7 deficiency reveals the significance of the cuticular plate for hearing function. Nat. Commun..

[19] Haag N., Schuler S., Nietzsche S., Hubner C.A., Strenzke N., Qualmann B., Kessels M.M. (2018). The Actin Nucleator Cobl Is Critical for Centriolar Positioning, Postnatal Planar Cell Polarity Refinement, and Function of the Cochlea. Cell Rep..

[20] Zhu G.J., Huang Y., Zhang L., Yan K., Qiu C., He Y., Liu Q., Zhu C., Morin M., Moreno-Pelayo M.A. (2023). Cingulin regulates hair cell cuticular plate morphology and is required for hearing in human and mouse. EMBO Mol. Med..

[21] Chatterjee P., Morgan C.P., Krey J.F., Benson C., Goldsmith J., Bateschell M., Ricci A.J., Barr-Gillespie P.G. (2023). GIPC3 couples to MYO6 and PDZ domain proteins, and shapes the hair cell apical region. J. Cell Sci..

[22] Yao Q., Wang H., Chen H., Li Z., Jiang Y., Li Z., Wang J., Xing Y., Liu F., Yu D. (2022). Essential Role of *Sptan1* in Cochlear Hair Cell Morphology and Function Via Focal Adhesion Signaling. Mol. Neurobiol..

[23] Pollock L.M., McDermott B.M. (2015). The cuticular plate: A riddle, wrapped in a mystery, inside a hair cell. Birth Defects Res. Part C Embryo Today Rev..

[24] Tilney L.G., Tilney M.S., DeRosier D.J. (1992). Actin filaments, stereocilia, and hair cells: How cells count and measure. Annu. Rev. Cell Biol..

[25] Anniko M. (1983). Cytodifferentiation of cochlear hair cells. Am. J. Otolaryngol..

[26] Nishida Y., Rivolta M.N., Holley M.C. (1998). Timed markers for the differentiation of the cuticular plate and stereocilia in hair cells from the mouse inner ear. J. Comp. Neurol..

[27] Szarama K.B., Gavara N., Petralia R.S., Kelley M.W., Chadwick R.S. (2012). Cytoskeletal changes in actin and microtubules underlie the developing surface mechanical properties of sensory and supporting cells in the mouse cochlea. Development.

[28] Etournay R., Lepelletier L., Boutet de Monvel J., Michel V., Cayet N., Leibovici M., Weil D., Foucher I., Hardelin J.P., Petit C. (2010). Cochlear outer hair cells undergo an apical circumference remodeling constrained by the hair bundle shape. Development.

[29] Sumi-Ichinose C., Ichinose H., Metzger D., Chambon P. (1997). SNF2beta-BRG1 is essential for the viability of F9 murine embryonal carcinoma cells. Mol. Cell. Biol..

[30] Yang H., Xie X., Deng M., Chen X., Gan L. (2010). Generation and characterization of Atoh1-Cre knock-in mouse line. Genesis.

[31] Jin Y., Ren N., Li S., Fu X., Sun X., Men Y., Xu Z., Zhang J., Xie Y., Xia M. (2016). Deletion of Brg1 causes abnormal hair cell planer polarity, hair cell anchorage, and scar formation in mouse cochlea. Sci. Rep..

[32] Legendre K., Safieddine S., Kussel-Andermann P., Petit C., El-Amraoui A. (2008). αII-βV spectrin bridges the plasma membrane and cortical lattice in the lateral wall of the auditory outer hair cells. J. Cell Sci..

[33] Cao H., Yin X., Cao Y., Jin Y., Wang S., Kong Y., Chen Y., Gao J., Heller S., Xu Z. (2013). FCHSD1 and FCHSD2 are expressed in hair cell stereocilia and cuticular plate and regulate actin polymerization in vitro. PLoS ONE.

[34] Raphael Y., Athey B.D., Wang Y., Lee M.K., Altschuler R.A. (1994). F-actin, tubulin and spectrin in the organ of Corti: Comparative distribution in different cell types and mammalian species. Hear. Res..

[35] Tarchini B., Jolicoeur C., Cayouette M. (2013). A molecular blueprint at the apical surface establishes planar asymmetry in cochlear hair cells. Dev. Cell.

[36] Liberman M.C. (1987). Chronic ultrastructural changes in acoustic trauma: Serial-section reconstruction of stereocilia and cuticular plates. Hear. Res..

[37] Steyger P.S., Furness D.N., Hackney C.M., Richardson G.P. (1989). Tubulin and microtubules in cochlear hair cells: Comparative immunocytochemistry and ultrastructure. Hear. Res..

[38] Li L., Yang X.J. (2015). Tubulin acetylation: Responsible enzymes, biological functions and human diseases. Cell. Mol. Life Sci..

[39] Ezan J., Lasvaux L., Gezer A., Novakovic A., May-Simera H., Belotti E., Lhoumeau A.C., Birnbaumer L., Beer-Hammer S., Borg J.P. (2013). Primary cilium migration depends on G-protein signalling control of subapical cytoskeleton. Nat. Cell Biol..

[40] Bhonker Y., Abu-Rayyan A., Ushakov K., Amir-Zilberstein L., Shivatzki S., Yizhar-Barnea O., Elkan-Miller T., Tayeb-Fligelman E., Kim S.M., Landau M. (2016). The GPSM2/LGN GoLoco motifs are essential for hearing. Mamm. Genome.

[41] Beer-Hammer S., Lee S.C., Mauriac S.A., Leiss V., Groh I.A.M., Novakovic A., Piekorz R.P., Bucher K., Chen C., Ni K. (2018). Galphai Proteins are Indispensable for Hearing. Cell. Physiol. Biochem..

[42] Jarysta A., Tadenev A.L.D., Day M., Krawchuk B., Low B.E., Wiles M.V., Tarchini B. (2024). Inhibitory G proteins play multiple roles to polarize sensory hair cell morphogenesis. eLife.

[43] Morin X., Bellaiche Y. (2011). Mitotic spindle orientation in asymmetric and symmetric cell divisions during animal development. Dev. Cell.

[44] Tadenev A.L.D., Akturk A., Devanney N., Mathur P.D., Clark A.M., Yang J., Tarchini B. (2019). GPSM2-GNAI Specifies the Tallest Stereocilia and Defines Hair Bundle Row Identity. Curr. Biol..

[45] Tarchini B., Tadenev A.L., Devanney N., Cayouette M. (2016). A link between planar polarity and staircase-like bundle architecture in hair cells. Development.

[46] Shi Y., Lin L., Wang C., Zhu J. (2022). Promotion of row 1-specific tip complex condensates by Gpsm2-Galphai provides insights into row identity of the tallest stereocilia. Sci. Adv..

[47] Mauriac S.A., Hien Y.E., Bird J.E., Carvalho S.D., Peyroutou R., Lee S.C., Moreau M.M., Blanc J.M., Geyser A., Medina C. (2017). Defective Gpsm2/Galphai3 signalling disrupts stereocilia development and growth cone actin dynamics in Chudley-McCullough syndrome. Nat. Commun..

[48] Dogterom M., Koenderink G.H. (2019). Actin-microtubule crosstalk in cell biology. Nat. Rev. Mol. Cell Biol..

[49] Fletcher D.A., Mullins R.D. (2010). Cell mechanics and the cytoskeleton. Nature.

[50] Gale J.E., Ashmore J.F. (1997). An intrinsic frequency limit to the cochlear amplifier. Nature.

[51] Issa N.P., Hudspeth A.J. (1996). The entry and clearance of Ca2+ at individual presynaptic active zones of hair cells from the bullfrog’s sacculus. Proc. Natl. Acad. Sci. USA.

[52] Liberman M.C., Gao J., He D.Z., Wu X., Jia S., Zuo J. (2002). Prestin is required for electromotility of the outer hair cell and for the cochlear amplifier. Nature.

[53] Yu X.L., Lewis E.R., Feld D. (1991). Seismic and auditory tuning curves from bullfrog saccular and amphibian papillar axons. J. Comp. Physiol. A Sens. Neural Behav. Physiol..

[54] Rudnicki M., Schoppe O., Isik M., Volk F., Hemmert W. (2015). Modeling auditory coding: From sound to spikes. Cell Tissue Res..

[55] Fettiplace R. (2017). Hair Cell Transduction, Tuning, and Synaptic Transmission in the Mammalian Cochlea. Compr. Physiol..

